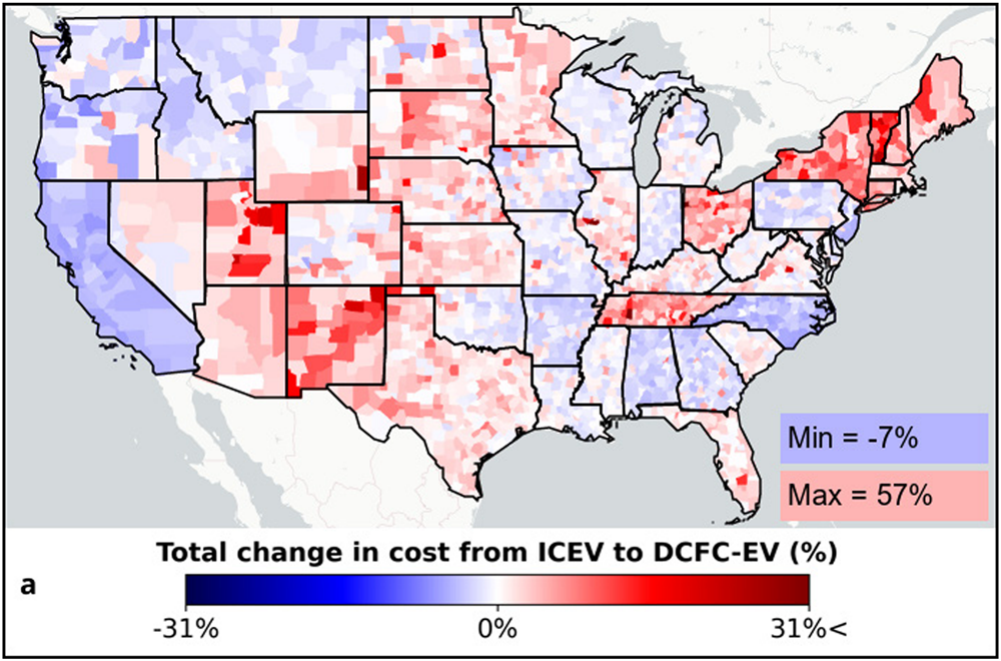# Author Correction: Comparing costs and climate impacts of various electric vehicle charging systems across the United States

**DOI:** 10.1038/s41467-024-49525-1

**Published:** 2024-06-18

**Authors:** Noah Horesh, David A. Trinko, Jason C. Quinn

**Affiliations:** Department of Mechanical Engineering, Colorado State University, 1374 Campus Delivery, Fort Collins, CO USA

**Keywords:** Energy economics, Environmental impact

Correction to: *Nature Communications* 10.1038/s41467-024-49157-5, published online 01 June 2024

In this article, the legend for Figure 1a read ‘Total change in cost from ICEV to BSS-EV’ and should have been ‘Total change in cost from ICEV to DCFC-EV’. The original article has been corrected.