# Supplementary material for: Comparing costs and climate impacts of various electric vehicle charging systems across the United States
Source: Nat Commun. 2024 Jun 1;15:4680. doi: 10.1038/s41467-024-49157-5 (PMC11144208; doi:10.1038/s41467-024-49157-5)
Supplement: Supplementary file 1 — Supplementary Information [file 41467_2024_49157_MOESM1_ESM.pdf]

Supplementary Information for

Comparing costs and climate impacts of various electric vehicle charging systems across the United States

Noah Horesh[1], David Trinko[1], Jason C. Quinn[1]*

[1]Department of Mechanical Engineering, Colorado State University; Fort Collins, 80524, United States.

*Corresponding author: Jason.Quinn@colostate.edu

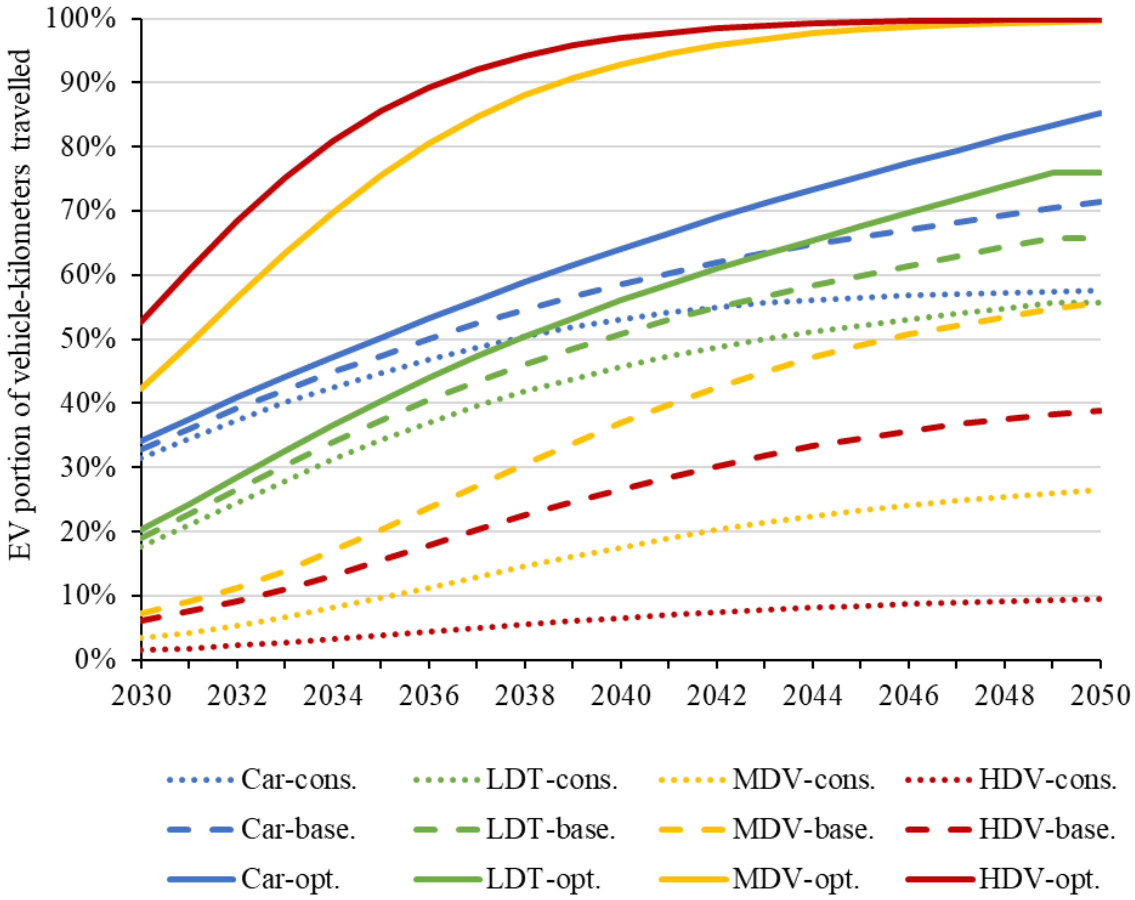

Supplementary Figure 1. Electric vehicle (EV) adoption scenarios for modeled vehicles. Optimistic (Opt.), baseline (Base.), and conservative (Cons.) adoption rates are presented for electric passenger cars (Car), light-duty trucks (LDT), medium-duty vehicles (MDV), and heavy-duty vehicles (HDV). Adapted from Mai et al. (2018)[1] and Konstantinou and Gkritza (2023)[2]. Source data are provided as a Source Data file.

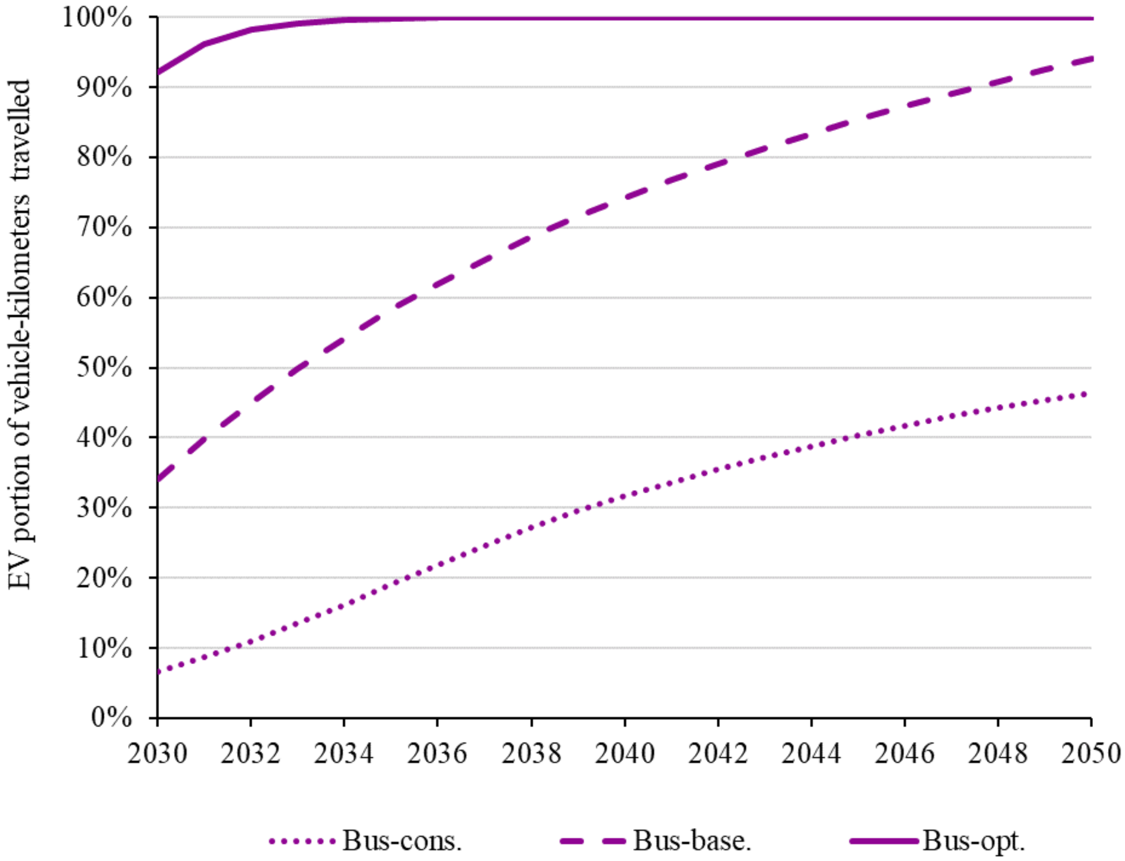

Supplementary Figure 2. Electric vehicle adoption scenarios for buses. Adoption curves are presented for optimistic (Opt.), baseline (Base.), and conservative (Cons.) scenarios. Adapted from Mai et al. (2018)[1]. Source data are provided as a Source Data file.

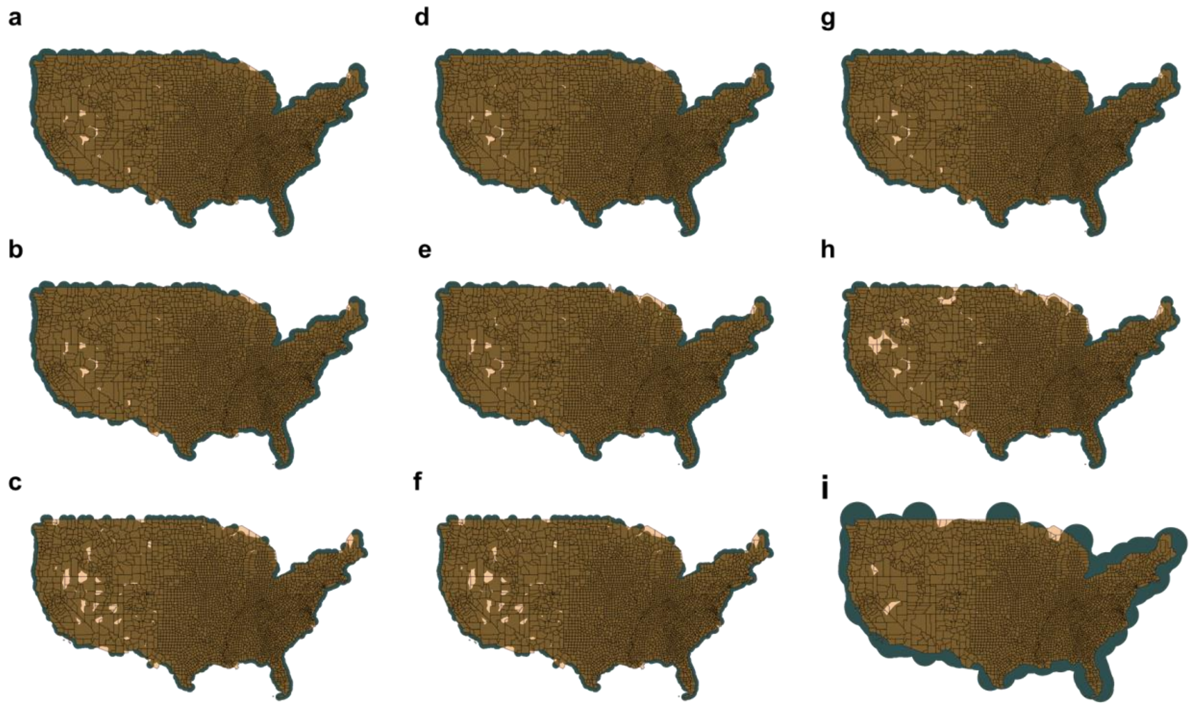

Supplementary Figure 3. Charging infrastructure deployment coverage. Coverage is shown by the darkened color for (**a-c**) optimistic, (**d-f**) baseline, and (**g-i**) conservative electric vehicle adoption scenarios with deployment for (**a, d, g**) Direct Current Fast Charging, (**b, e, h**) Battery Swapping, and (**c, f, i**) Dynamic Wireless Power Transfer. A coverage radius of 80 kilometers (50 miles) is used for each Direct Current Fast Charging and Battery Swapping site. Dynamic Wireless Power Transfer roads have a 56 kilometer (35-mile) radius for optimistic and baseline deployment scenarios. The radius for conservative Dynamic Wireless Power Transfer roadways is 214 kilometers (133 miles), which represents the shortest range among electric vehicle categories. Base map layer is available from OpenStreetMap (openstreetmap.org/copyright).

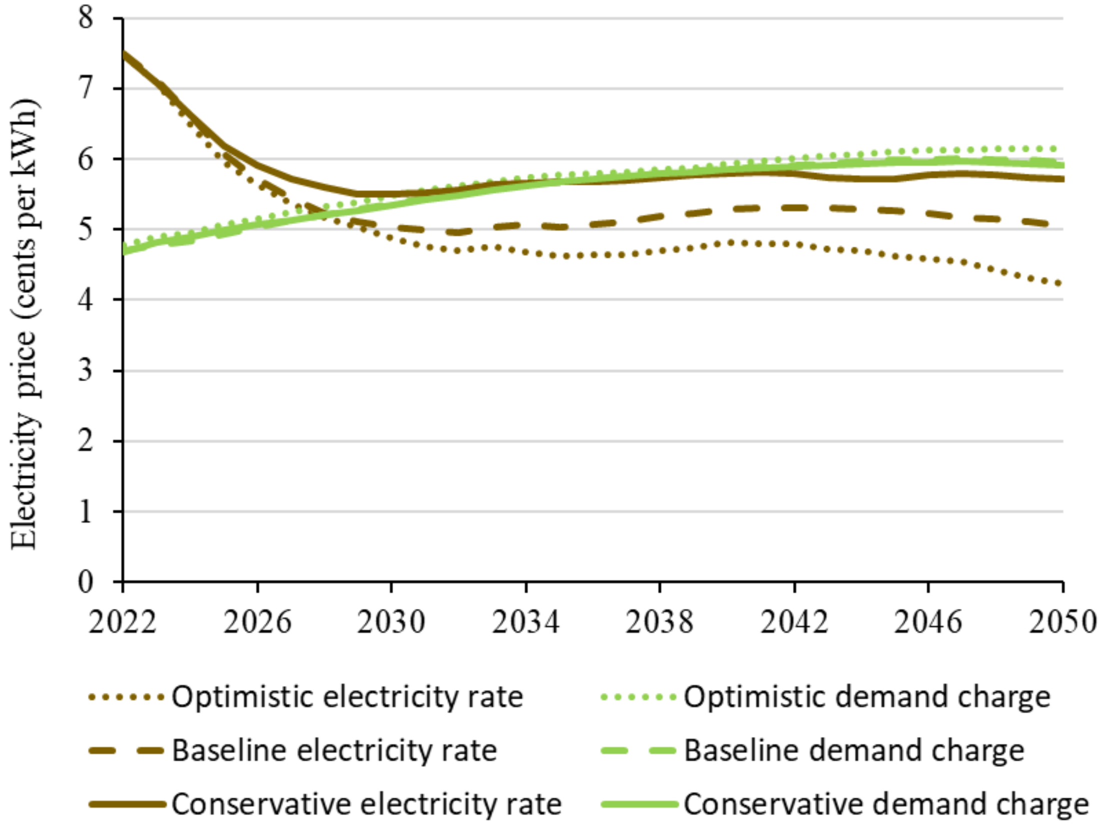

Supplementary Figure 4. Forecasted electricity prices from 2022 to 2050. Optimistic, baseline, and conservative scenarios are presented for a set of electricity rates and corresponding demand charges. Adapted from Annual Energy Outlook (2023)[3]. Source data are provided as a Source Data file.

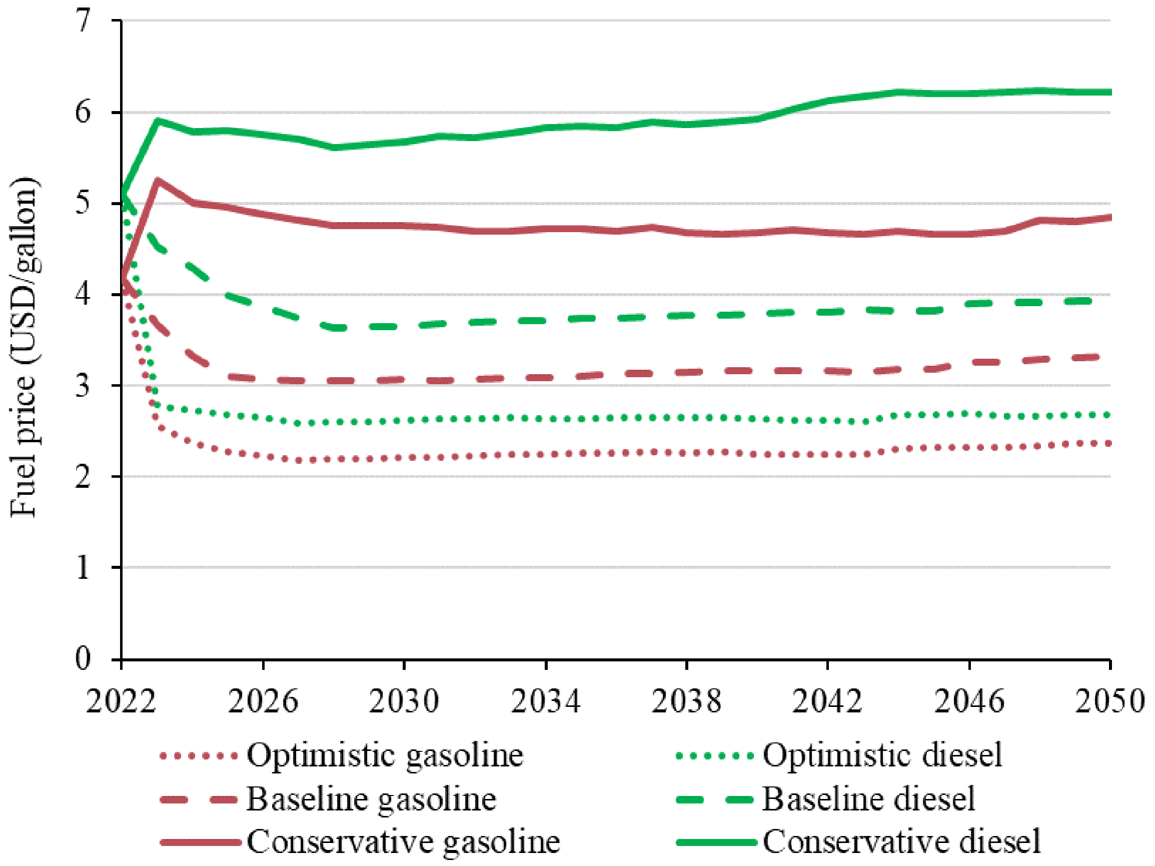

Supplementary Figure 5. Forecasted fuel prices from 2022 to 2050. Optimistic, baseline, and conservative scenarios are presented for gasoline and diesel fuel. Adapted from Annual Energy Outlook (2023)[3]. Abbreviations: 2022 United States Dollars (USD). Source data are provided as a Source Data file.

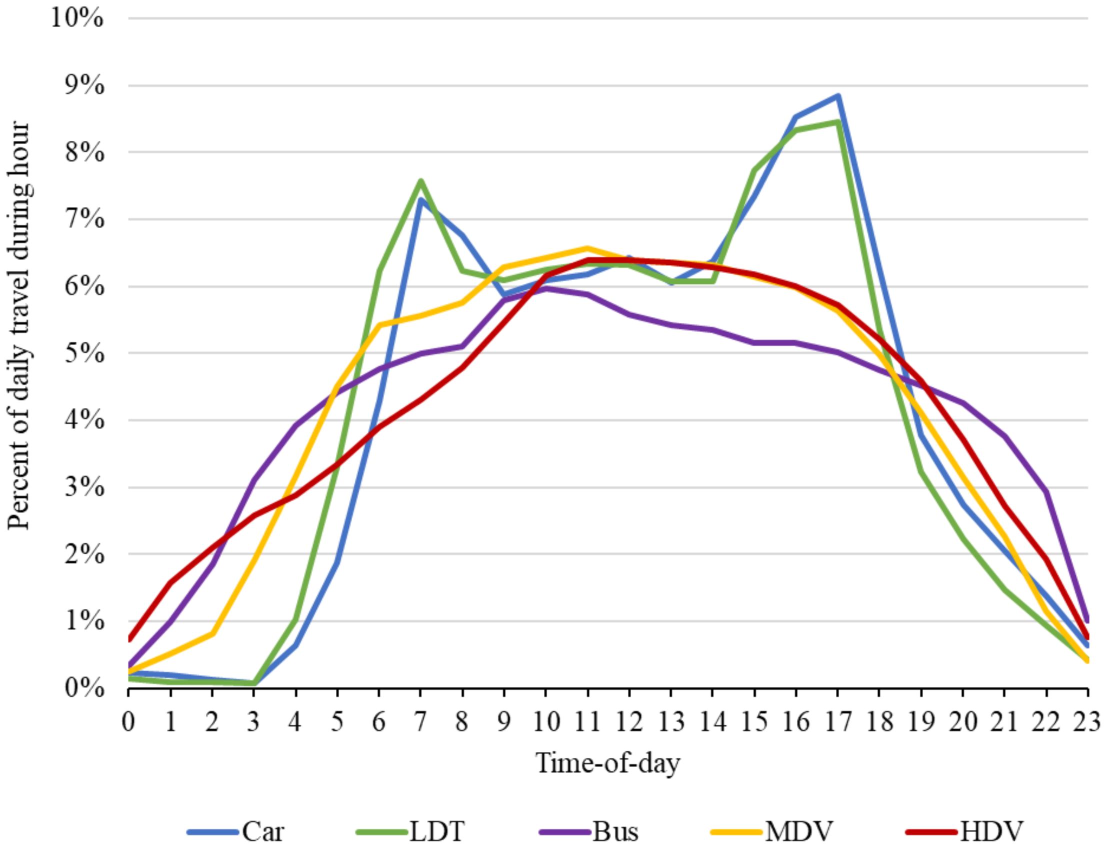

Supplementary Figure 6. Portion of daily vehicle travel during each hour of the day. Results are shown for passenger cars (Car), light-duty trucks (LDT), buses (Bus), medium-duty vehicles (MDV), and heavy-duty vehicles (HDV). Source data are provided as a Source Data file.

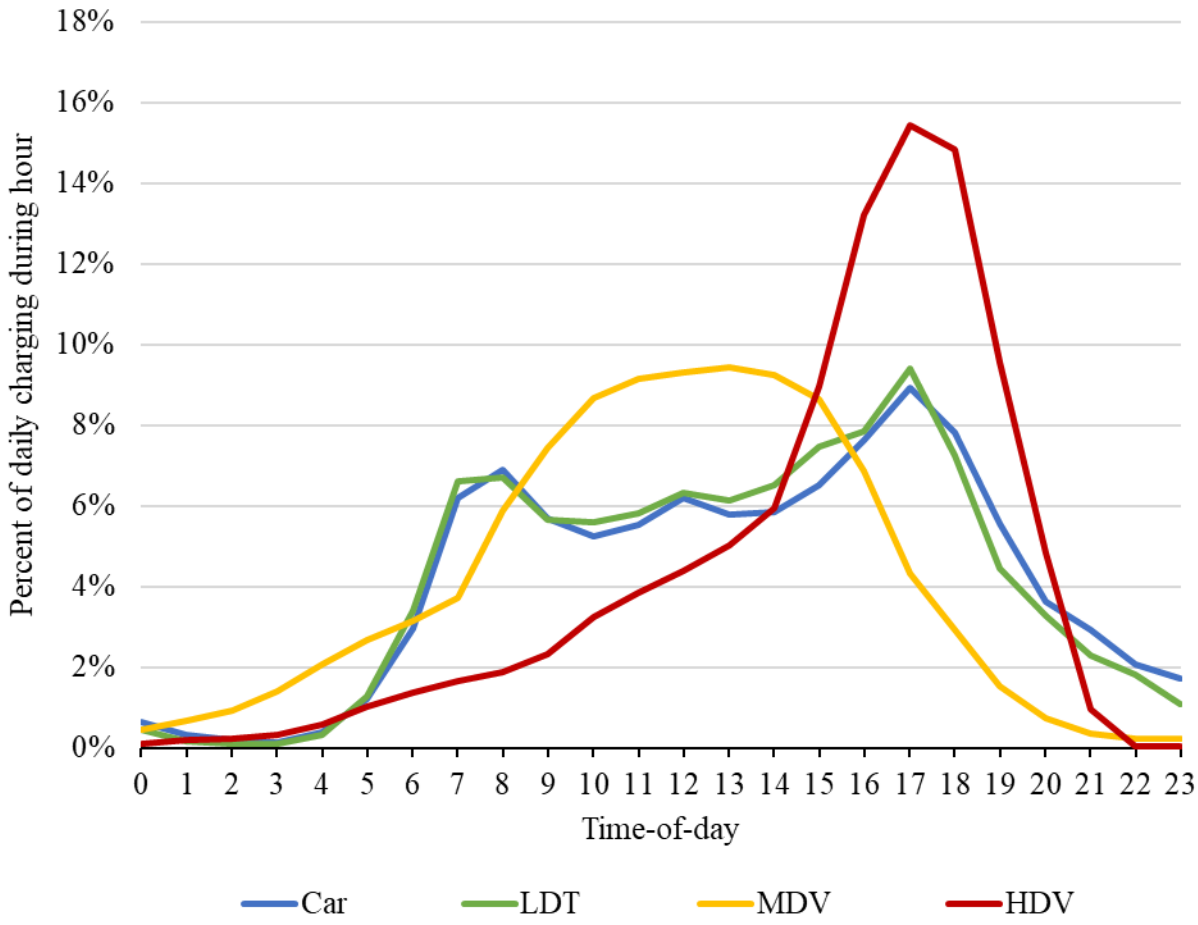

Supplementary Figure 7. Portion of daily direct current fast charging during each hour of the day. Results are shown for passenger cars (Car), light-duty trucks (LDT), medium-duty vehicles (MDV), and heavy-duty vehicles (HDV). Source data are provided as a Source Data file.

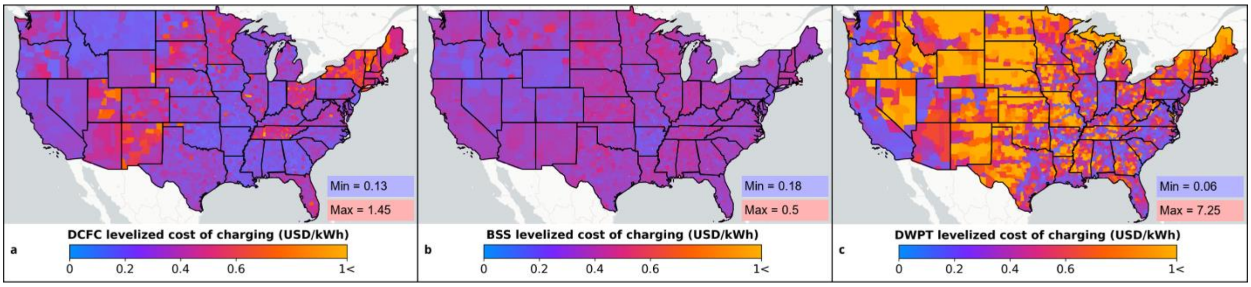

Supplementary Figure 8. Levelized cost of charging in United States counties. Results are presented for **a** Direct Current Fast Charging (DCFC), **b** Battery Swapping (BSS), and **c** Dynamic Wireless Power Transfer (DWPT). The baseline scenarios are shown in this figure with all scenarios shown in the repository. Abbreviations: 2022 United States Dollars (USD); kilowatt-hour (kWh). Source data are provided as a Source Data file and base map layer is available from OpenStreetMap (openstreetmap.org/copyright).

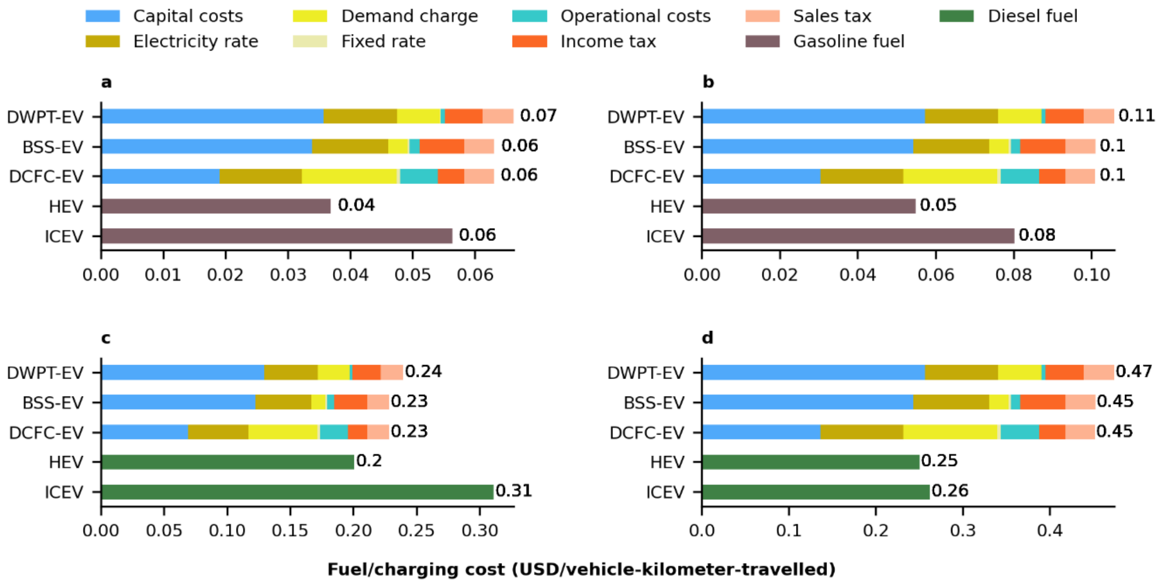

Supplementary Figure 9. Breakdown of the charging/fuel cost. Average costs in the contiguous United States are shown for electric vehicles charged via Direct Current Fast Charging (DCFC-EV), Battery Swapping (BSS-EV), and Dynamic Wireless Power Transfer (DWPT-EV). Results are compared to an average internal combustion engine vehicle (ICEV) and hybrid electric vehicle (HEV) fueled by gasoline or diesel for the vehicle categories of **a** passenger car, **b** light duty truck, **c** medium duty vehicle, and **d** heavy duty vehicle. The baseline scenarios are shown in this figure with all scenarios shown in the repository. Abbreviations: 2022 United States Dollars (USD). Source data are provided as a Source Data file.

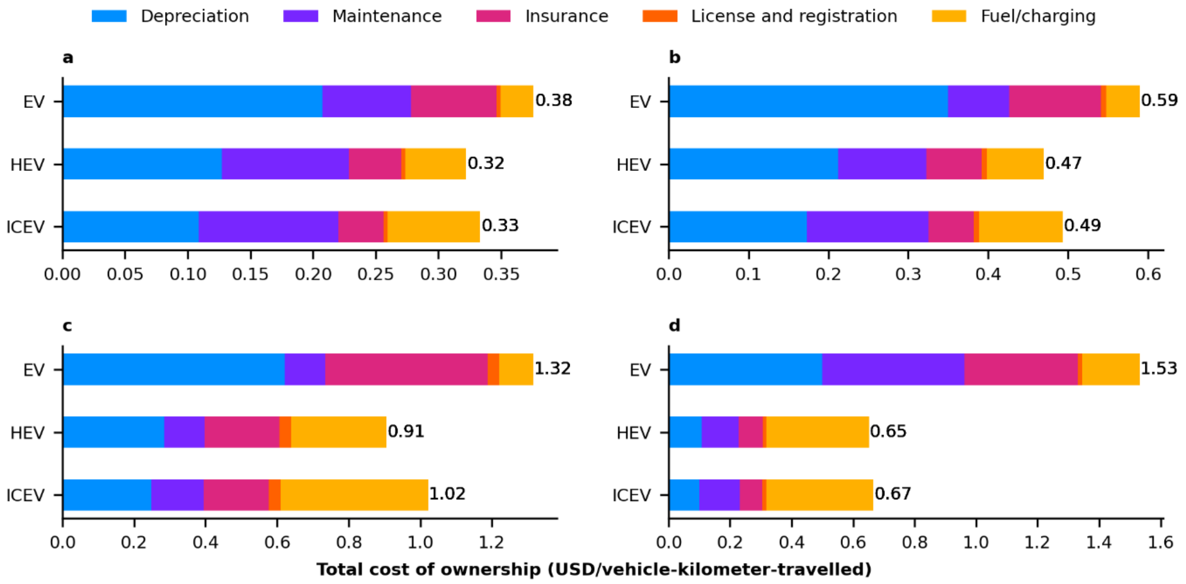

Supplementary Figure 10. Breakdown of the 10-year total cost of ownership in 2022. Results are presented for an average **a** passenger car, **b** light duty truck, **c** medium duty vehicle, and **d** heavy duty vehicle in the contiguous United States. The vehicle types include an average electric vehicle (EV), internal combustion engine vehicle (ICEV), and hybrid electric vehicle (HEV) from each vehicle category. Abbreviations: 2022 United States Dollars (USD). Source data are provided as a Source Data file.

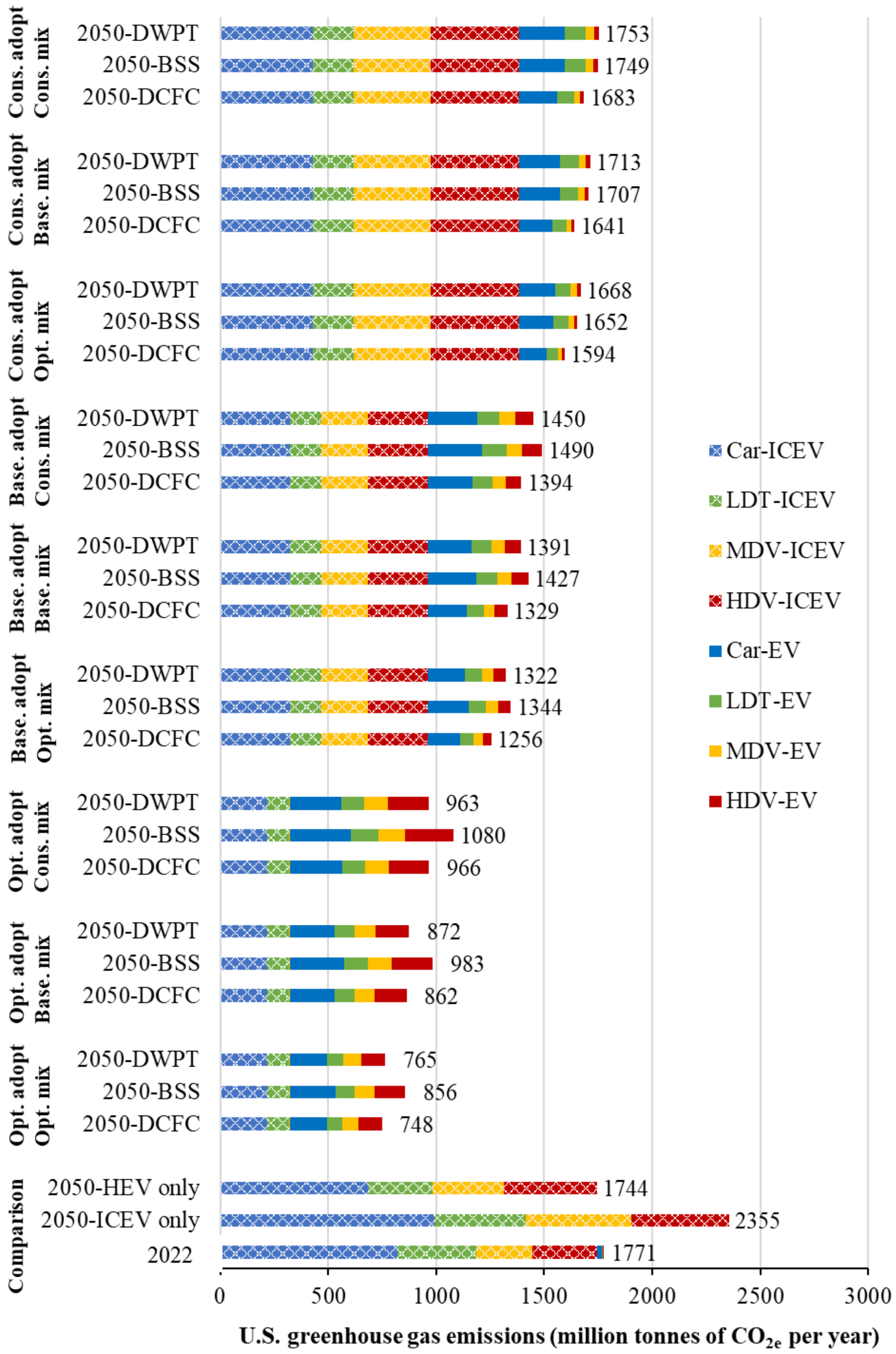

Supplementary Figure 11. Breakdown of the total vehicle greenhouse gas emissions in 2022 and 2050. The emissions are presented for optimistic (Opt.), baseline (Base.), and conservative (Cons.) electric vehicle adoption (adopt) and electricity mix (mix) scenarios. Vehicle emissions from the contiguous United States (U.S.) are shown for electric vehicles (EVs) charged via Direct Current Fast Charging (DCFC), Battery Swapping (BSS), and Dynamic Wireless Power Transfer (DWPT). Results are compared to an average internal combustion engine vehicle (ICEV) and hybrid electric vehicle (HEV). The results are broken down for passenger cars (Car), light-duty trucks (LDT), medium-duty vehicles (MDV), and heavy-duty vehicles (HDV). Abbreviations: Carbon Dioxide equivalent (CO_{2e}). Source data are provided as a Source Data file.

Supplementary Table 1. State level income and sales tax rates. Sales taxes rates are an average of the combined county and state rates.

State	Income tax[4]	Sales tax[5]
Alabama	6.5%	9.2%
Arizona	4.9%	8.4%
Arkansas	5.3%	9.5%
California	8.8%	8.8%
Colorado	4.6%	7.8%
Connecticut	7.5%	6.4%
Delaware	8.7%	0.0%
District of Columbia	8.3%	6.0%
Florida	5.5%	7.0%
Georgia	5.8%	7.3%
Idaho	5.8%	6.0%
Illinois	9.5%	8.8%
Indiana	4.9%	7.0%
Iowa	5.5%	6.9%
Kansas	4.0%	8.7%
Kentucky	5.0%	6.0%
Louisiana	3.5%	9.5%
Maine	3.5%	5.5%
Maryland	8.3%	6.0%
Massachusetts	8.0%	6.3%
Michigan	6.0%	6.0%
Minnesota	9.8%	7.5%
Mississippi	5.0%	7.1%
Missouri	4.0%	8.3%
Montana	6.8%	0.0%
Nebraska	5.6%	6.9%
Nevada	0.0%	8.2%
New Hampshire	7.5%	0.0%
New Jersey	6.5%	6.6%
New Mexico	4.8%	7.8%
New York	6.5%	8.5%
North Carolina	2.5%	7.0%
North Dakota	3.6%	7.0%
Ohio	0.0%	7.2%
Oklahoma	4.0%	9.0%
Oregon	6.6%	0.0%
Pennsylvania	9.0%	6.3%
Rhode Island	7.0%	7.0%
South Carolina	5.0%	7.4%
South Dakota	0.0%	6.4%
Tennessee	6.5%	9.5%
Texas	0.0%	8.2%
Utah	4.9%	7.2%
Vermont	8.5%	6.2%
Virginia	6.0%	5.8%
Washington	0.0%	9.3%
West Virginia	6.5%	6.5%
Wisconsin	7.9%	5.4%
Wyoming	0.0%	5.2%

Supplementary Table 2. Breakdown of the baseline scenario's urban roadway civil cost for dynamic wireless power transfer. The values are in 2022 United States Dollars (USD). Abbreviations: kilometer (km).

Parameter	Value (thousand USD per lane-km)
Electrical	727[6]
Pavement removal	106[6]
Traffic control	112[6]
Signage	12[6]
Soft cost	820[6]
Pavement	552[6]
Install materials	69[6]
Subtotal	2398
Contingency	10%[6]
Total	2638

Supplementary Table 3. Average gasoline and diesel fuel prices from 2022 in United States Dollars (USD).

Parameter	Regular gasoline (USD/gallon)[7]	Diesel (USD/gallon)[7]
Alabama	3.121	4.148
Arkansas	3.091	3.975
Arizona	3.939	4.439
California	4.924	5.598
Colorado	3.938	4.668
Connecticut	3.322	4.668
Delaware	3.263	4.105
District of Columbia	3.511	4.734
Florida	3.325	4.416
Georgia	3.263	4.292
Iowa	3.25	3.935
Idaho	3.679	4.489
Illinois	3.658	4.306
Indiana	3.404	4.456
Kansas	3.091	3.918
Kentucky	3.17	4.127
Louisiana	3.1	4.05
Massachusetts	3.3	4.757
Maryland	3.303	4.217
Maine	3.429	5.179
Michigan	3.548	4.421
Minnesota	3.32	4.052
Missouri	3.056	3.904
Mississippi	3.018	4.026
Montana	3.274	4.219
North Carolina	3.256	4.174
North Dakota	3.276	4.327
Nebraska	3.291	4.043
New Hampshire	3.264	4.777
New Jersey	3.304	4.32
New Mexico	3.424	4.162
Nevada	4.344	4.694
New York	3.466	4.996
Ohio	3.41	4.319
Oklahoma	3.077	3.814
Oregon	3.91	4.714
Pennsylvania	3.634	4.841
Rhode Island	3.292	4.713
South Carolina	3.18	4.071
South Dakota	3.346	4.144
Tennessee	3.159	4.078
Texas	3.079	3.868
Utah	3.85	4.433
Virginia	3.236	4.273
Vermont	3.443	4.591
Washington	4.247	5.027
Wisconsin	3.338	3.937
West Virginia	3.421	4.442
Wyoming	3.433	4.526

Supplementary Table 4. Annual license and registration costs. Costs are for cars, light-duty trucks (LDTs), medium-duty vehicles (MDVs), and heavy-duty vehicles (HDVs) in United States Dollars (USD).

State	Car (USD)[8]	LDT (USD)[8]	MDV (USD)[8]	HDV (USD)[8]
Alabama	50	50	586	816
Arizona	160	205	210	930
Arkansas	20	24	130	1350
California	133	378	1325	2119
Colorado	173	198	1788	2105
Connecticut	178	176	281	1520
Delaware	40	40	877	709
District of Columbia	72	155	125	340
Florida	23	33	589	995
Georgia	20	20	365	400
Idaho	53	51	517	3202
Illinois	151	151	1698	2790
Indiana	21	30	811	1351
Iowa	153	214	1060	1695
Kansas	43	52	132	1727
Kentucky	21	21	704	1445
Louisiana	10	30	348	494
Maine	35	35	638	835
Maryland	135	162	596	1288
Massachusetts	30	30	840	1200
Michigan	97	141	975	1277
Minnesota	55	55	865	1760
Mississippi	29	21	1663	2872
Missouri	57	57	63	1720
Montana	115	102	415	315
Nebraska	21	21	138	933
Nevada	33	33	986	1360
New Hampshire	31	43	557	1240
New Jersey	49	69	162	841
New Mexico	40	55	172	172
New York	24	39	334	968
North Carolina	39	57	871	963
North Dakota	66	126	632	1059
Ohio	36	36	740	1351
Oklahoma	81	79	653	954
Oregon	132	132	220	320
Pennsylvania	38	38	882	1688
Rhode Island	48	58	140	1044
South Carolina	40	40	844	1600
South Dakota	65	95	873	1311
Tennessee	27	27	898	1334
Texas	51	51	180	840
Utah	77	68	420	660
Vermont	76	76	271	1910
Virginia	31	36	600	980
Washington	68	88	771	1832
West Virginia	52	52	755	980
Wisconsin	85	100	1135	2578
Wyoming	183	240	60	60

Supplementary Table 5. Annual insurance costs for a car, light-duty truck (LDT), medium-duty vehicle (MDV), and heavy-duty vehicle (HDV). Cost is per thousand United States Dollars (USD) of vehicle value.

State	Car (USD)[8]	LDT (USD)[8]	MDV (USD)[8]	HDV (USD)[8]
Alabama	43.00	43.00	59.00	61.75
Arizona	35.75	35.75	35.00	51.25
Arkansas	53.25	53.25	63.00	64.75
California	53.75	53.75	68.00	61.00
Colorado	50.75	50.75	42.00	52.50
Connecticut	40.50	40.50	97.25	73.75
Delaware	39.25	39.25	99.75	85.25
District of Columbia	36.00	36.00	55.00	56.75
Florida	39.25	39.25	74.00	84.50
Georgia	43.75	43.75	87.25	89.50
Idaho	38.00	38.00	39.50	39.00
Illinois	40.75	40.75	44.25	59.75
Indiana	36.00	36.00	48.50	48.25
Iowa	40.75	40.75	32.25	38.50
Kansas	57.25	57.25	38.25	44.00
Kentucky	58.75	58.75	66.25	67.25
Louisiana	53.00	53.00	113.25	101.75
Maine	29.50	29.50	54.75	59.75
Maryland	35.50	35.50	63.75	69.50
Massachusetts	34.75	34.75	31.25	73.75
Michigan	66.50	66.50	51.25	59.75
Minnesota	39.75	39.75	55.50	55.50
Mississippi	45.75	45.75	20.50	20.25
Missouri	54.25	54.25	44.00	51.00
Montana	52.75	52.75	37.25	41.25
Nebraska	49.75	49.75	36.00	37.50
Nevada	43.00	43.00	61.25	73.25
New Hampshire	32.50	32.50	39.25	49.50
New Jersey	32.25	32.25	119.25	87.75
New Mexico	38.50	38.50	42.00	42.25
New York	41.25	41.25	101.00	73.50
North Carolina	26.50	26.50	42.75	46.00
North Dakota	50.00	50.00	37.00	40.00
Ohio	31.75	31.75	40.75	43.00
Oklahoma	55.75	55.75	53.75	58.00
Oregon	34.75	34.75	48.75	48.75
Pennsylvania	44.75	44.75	43.25	54.00
Rhode Island	51.75	51.75	80.75	74.75
South Carolina	36.25	36.25	54.00	58.00
South Dakota	68.25	68.25	38.50	39.00
Tennessee	55.00	55.00	55.00	59.75
Texas	41.75	41.75	60.50	62.75
Utah	31.50	31.50	52.50	48.25
Vermont	39.25	39.25	39.75	41.75
Virginia	30.75	30.75	57.25	57.00
Washington	32.25	32.25	48.75	55.00
West Virginia	46.00	46.00	67.25	63.50
Wisconsin	39.75	39.75	38.50	40.00
Wyoming	56.25	56.25	28.25	31.00

Supplementary Table 6. Breakdown of Direct Current Fast Charging infrastructure emissions. Abbreviations: Carbon Dioxide equivalent (CO_{2e}), metric tonne (t), kilowatt (kW).

Parameter	Value (t-CO_{2e}/charger)
Charger pedestal	7.1[9,10]
150-kW power cabinet	2.4 (Supplementary Table 7)
350-kW power cabinets	4.9 (Supplementary Table 7)
Implementation	7.9[11]
Construction	1.4[12]
Total 150-kW	19
Total 350-kW	21

Supplementary Table 7. Breakdown of one power cabinet's greenhouse gas (GHG) emissions intensity. Abbreviations: Carbon Dioxide equivalent (CO_{2e}), kilogram (kg).

Material	Emissions rate (kg-CO_{2e}/kg)	Weight (kg)	Total GHG-intensity (kg-CO_{2e})
Steel	1.1 [9]	847	915
Copper	0.82 [9]	327	267
Fiberglass	8.8 [9]	45	400
Aluminum	7.3 [9]	115	844
Ferrite	2.0 [9]	6	12
Total		1340	2439

Supplementary Table 8. Breakdown of Battery Swapping (BSS) infrastructure emissions. Abbreviations: Carbon Dioxide equivalent (CO_{2e}), kilogram (kg), kilowatt-hour (kWh), kilowatt (kW), light-duty truck (LDT), medium-duty vehicle (MDV), and heavy-duty vehicle (HDV).

Parameter	Value	Units
7.7-kW Charger pedestal	312[9,13]	kg-CO_{2e}/charger
50-kW Charger pedestal	4673[9,14]	kg-CO_{2e}/charger
7.7-kW construction	200[12]	kg-CO_{2e}/charger
50-kW construction	1380[12]	kg-CO_{2e}/charger
Automated supply and retrieval system	346[9]	kg-CO_{2e}/BSS
Building	36[9]	kg-CO_{2e}/square-meter
Car battery	5348[15]	kg-CO_{2e}/battery
LDT battery	8961[15]	kg-CO_{2e}/battery
MDV battery	10694[15]	kg-CO_{2e}/battery
HDV battery	36865[15]	kg-CO_{2e}/battery
Battery cabinet	270[9,16]	kg-CO_{2e}/kWh

Supplementary Table 9. Breakdown of Dynamic Wireless Power Transfer infrastructure emissions. Abbreviations: Carbon Dioxide equivalent (CO_{2e}), metric tonne (t), kilometer (km).

Parameter	Value (t-CO_{2e}/lane-km)
Electronic components [17]	
Transformer	18.58[9]
AC/DC converter	16.10[9]
Shelter	0.11[9]
Super-capacitors box	0.01[9]
Control power supply	0.38[9]
Coil	2.90[9]
Connectors	0.06[9]
Capacitors	2.06[9]
Power electronics board	0.53[9]
Housing	0.55[9]
Connectors	0.06[9]
Distribution lines	25.19[9]
Pavement	
Concrete	3388.35[6,9]
Reinforcement bars	63.20[6,9]
Transportation	
Electronic components	0.54[9,17]
Pavement	21.19[9,17]
Equipment	0.43[9,17]
Construction	
Milling	0.36[9,17]
Sweeping	0.02[9,17]
Paver	0.32[9,17]
Total	3540.95

Supplementary Table 10. Emissions factors for Cambium (2022) resources. All values are in kilograms of Carbon Dioxide equivalent per kilowatt-hour. Unknown values are either set equal to zero or equal to the value from other regions. Abbreviations: oil-gas-steam (o-g-s), natural gas combined cycle (gas-cc), natural gas combustion turbine (gas-ct), hydropower (hydro), onshore wind (wind-ons), offshore wind (wind-ofs), concentrating solar power (csp), utility scale photovoltaics (upv), behind-the-meter photovoltaics (distpv), pumped hydro storage (phs), bioenergy with carbon capture and storage (beccs), renewable energy combustion turbine (re-ct), coal with carbon capture and storage (coal-ccs), natural gas combined cycle with carbon capture and storage (gas-cc-ccs), Midwest Reliability Organization (MRO), Northeast Power Coordinating Council (NPCC), Reliability First Corporation (RFC), SERC Reliability Corporation (SERC), Texas Reliability Entity (TRE), Western Electricity Coordinating Council (WECC).

Cambium (2022) resource[18]	MRO[9]	NPCC[9]	RFC[9]	SERC[9]	TRE[9]	WECC[9]
nuclear	0.0066	0.0066	0.0066	0.0066	0.0066	0.0066
coal	1.25	1.24	1.21	1.28	1.23	1.23
o-g-s	1.27	1.18	1.22	1.03	1.10	1.86
gas-cc	0.44	0.44	0.43	0.46	0.43	0.42
gas-ct	0.68	0.66	0.64	0.74	0.64	0.60
hydro	0.0054	0.0054	0.0054	0.0054	0.0054	0.0054
geothermal	0.067	0.067	0.067	0.067	0.067	0.067
biomass	0.041	0.041	0.041	0.041	0.041	0.041
wind-ons	0.015	0.015	0.015	0.015	0.015	0.015
wind-ofs	0.016	0.016	0.016	0.016	0.016	0.016
csp	0.047	0.047	0.047	0.047	0.047	0.047
upv	0.068	0.068	0.068	0.068	0.068	0.056
distpv	0.071	0.071	0.071	0.071	0.070	0.059
phs	0.029	0.029	0.029	0.029	0.029	0.029
battery	0.0017	0.0017	0.0017	0.0017	0.0017	0.0017
beccs	0	0	0	0	0	0
re-ct	0	0	0	0	0	0
coal-ccs	0	0	0	0	0	0
gas-cc-ccs	0	0	0	0	0	0
canada	0.40	0.42	0	0	0	0.45

Supplementary References

1. Mai, T. T. *et al. Electrification Futures Study: Scenarios of Electric Technology Adoption and Power Consumption for the United States*. NREL/TP--6A20-71500, 1459351 http://www.osti.gov/servlets/purl/1459351/ (2018) doi:10.2172/1459351.
2. Konstantinou, T. & Gkritza, K. Are we getting close to truck electrification? U.S. truck fleet managers' stated intentions to electrify their fleets. *Transportation Research Part A: Policy and Practice* **173**, 103697 (2023).
3. Annual energy outlook. *U.S. Energy Information Administration* https://www.eia.gov/outlooks/aeo/data/browser/ (2023).
4. Fritts, J. State corporate income tax rates and brackets. *Tax Foundation* https://taxfoundation.org/publications/state-corporate-income-tax-rates-and-brackets/ (2023).
5. State sales tax rates. *Sales Tax Institute* https://www.salestaxinstitute.com/resources/rates.
6. Trinko, D. *et al.* Economic feasibility of in-motion wireless power transfer in a high-density traffic corridor. *eTransportation* **11**, 100154 (2022).
7. AAA Gas Prices. *AAA* https://gasprices.aaa.com/state-gas-price-averages/ (2022).
8. Burnham, A. *et al. Comprehensive Total Cost of Ownership Quantification for Vehicles with Different Size Classes and Powertrains*. ANL/ESD-21/4, 1780970, 167399 https://www.osti.gov/servlets/purl/1780970/ (2021) doi:10.2172/1780970.
9. Wernet, G. *et al.* The ecoinvent database version 3 (part I): Overview and methodology. *Int J Life Cycle Assess* **21**, 1218–1230 (2016).
10. Terra HP GEN 3 CE & UL markets - Leaflet. *ABB* https://search.abb.com/library/Download.aspx?DocumentID=9AKK107991A9632&LanguageCode=en&DocumentPartId=&Action=Launch (2023).
11. Lucas, A., Alexandra Silva, C. & Costa Neto, R. Life cycle analysis of energy supply infrastructure for conventional and electric vehicles. *Energy Policy* **41**, 537–547 (2012).

12. Mulrow, J. & Grubert, E. Greenhouse gas emissions embodied in electric vehicle charging infrastructure: a method and case study of Georgia, US 2021–2050. *Environ. Res.: Infrastruct. Sustain.* **3**, 015013 (2023).
13. Blink Charging Co. Level 2 AC EV IQ 200 charging stations | specifications. *Blink Charging* https://a-us.storyblok.com/f/1016941/x/e016f650d3/blink-charging-iq-200-specifications-sheet.pdf (2022).
14. Blink Charging Co. DCFC 50kW fast charger. *Blink Charging* https://blinkcharging.com/wp-content/uploads/2021/02/DCFC_Specs_Blink_50kw_75kW_175kW-1.pdf (2021).
15. Wang, M. *et al.* Greenhouse gases, regulated emissions, and energy use in technologies model ® (2022 .Net). Argonne National Laboratory https://doi.org/10.2172/1891644 (2022).
16. Ellingsen, L. A.-W. *et al.* Life cycle assessment of a lithium-ion battery vehicle pack. *Journal of Industrial Ecology* **18**, 113–124 (2014).
17. Marmiroli, B., Dotelli, G. & Spessa, E. Life cycle assessment of an on-road dynamic charging infrastructure. *Applied Sciences* **9**, 3117 (2019).
18. Gagnon, P., Cowiestoll, B. & Schwarz, M. *Cambium 2022 Scenario Descriptions and Documentation*. NREL/TP-6A40-84916, 1915250, MainId:85689 https://www.osti.gov/servlets/purl/1915250/ (2023) doi:10.2172/1915250.
